# Prediction of Chloride Diffusion Coefficient in Concrete Modified with Supplementary Cementitious Materials Using Machine Learning Algorithms

**DOI:** 10.3390/ma16031277

**Published:** 2023-02-02

**Authors:** Abdulrahman Fahad Al Fuhaid, Hani Alanazi

**Affiliations:** 1 Department of Civil and Environmental Engineering, College of Engineering, King Faisal University (KFU), P.O. Box 380, Al-Ahsa 31982, Saudi Arabia; 2 Department of Civil and Environmental Engineering, College of Engineering, Majmaah University, Al-Majmaah 11952, Saudi Arabia

**Keywords:** concrete, machine learning approach, chloride diffusion coefficient, durability

## Abstract

The chloride diffusion coefficient (Dcl) is one of the most important characteristics of concrete durability. This study aimed to develop a prediction model for the Dcl of concrete incorporating supplemental cementitious material. The datasets of concrete containing supplemental cementitious materials (SCMs) such as tricalcium aluminate (C_3_A), ground granulated blast furnace slag (GGBFS), and fly ash were used in developing the model. Five machine learning (ML) algorithms including adaptive neuro-fuzzy inference system (ANFIS), artificial neural network (ANN), support vector machine (SVM), and extreme learning machine (ELM) were used in the model development. The performance of the developed models was tested using five evaluation metrics, namely, normalized reference index (RI), coefficient of determination (R^2^), mean absolute error (MAE), and root mean square error (RMSE). The SVM models demonstrated the highest prediction accuracy with R^2^ values of 0.955 and 0.951 at the training and testing stage, respectively. The prediction accuracy of the machine learning (ML) algorithm was checked using the Taylor diagram and Boxplot, which confirmed that SVM is the best ML algorithm for estimating Dcl, thus, helpful in establishing reliable tools in concrete durability design.

## 1. Introduction

Due to its affordability and durability, reinforced concrete is considered the most frequently utilized material for constructing infrastructures. Nevertheless, the chloride-induced problem leads to the remarkable deterioration of concrete structures and maintenance costs, especially for structures located near or in coastal and offshore regions. Whenever chloride infiltrates the concrete and attains a high concentration around the reinforced steel, the reinforcements become damaged, thus corrosion occurs [1,2]. It is required to create highly impermeable concrete to restrict chloride ions from penetrating the concrete to prevent steel corrosion. As a result, understanding the quantifiable factors affecting the chloride penetration rate in concrete is necessary [3,4]. The performance technique considering the coefficient of chloride diffusion is among the three durability parameters for the design of reinforced concrete components exposed to marine locations [5,6]. Due to the time and resources needed, it is challenging to investigate the diffusion coefficient or coefficient infiltration in concrete for any concrete structure using experiments. Therefore, chloride penetration into the concrete analyzing approach such as the multispecies ionic transport model [7,8,9,10] and the geochemical model [1,2,11] needs the coefficient of chloride diffusion to develop models. Thus, to improve the concrete durability and lifespan of concrete structures and develop an effective maintenance strategy, it is essential to evaluate the Dcl in concrete. Numerous empirical models have been established to assess the Dcl, which include ordinary concrete and concretes containing SCMs such as fly ash and GGBFS [12,13]. However, the conventional prediction of the chloride diffusion mechanism is more complicated for concrete containing SCMs due to the complicated diffusion mechanism in concrete [14].

Due to the rapid development of AI technology over the past few decades, ML models have been utilized in several aspects of life [15,16]. Many civil engineering problems have been solved using ML algorithms. These include geotechnical engineering [17,18], pavement structures [19], structural engineering [20,21,22,23], composite structural elements [24], material science [25,26,27,28,29], and traffic engineering [30,31,32,33]. Moreover, previous research has been conducted to predict the Dcl in concrete using an AI-based approach [34,35,36]. Some of the algorithms used include multi-gene genetic programming [37], ensemble ML techniques, decision tree, multivariate adaptive regression spline [4,38,39], metaheuristic optimization algorithms [40], hybrid ANN models using three swarm-based optimization algorithms [41], three-phase composites model at mesoscopic levels [42], and a computational model [43]. Liu et al. [36] explored distinct features of the ANN model in developing a rational and robust prediction model for evaluating the Dcl of concrete. The network model was established using a reliable database from previous studies. The results showed that the ANN is a valuable algorithm for determining the inconsistencies in the dataset and is helpful for calculating the Dcl in concrete structures situated in an aggressive environment. To estimate the chloride penetration resistance of concrete containing groundwater pozzolan, rice husk ash, fly ash, and bottom ash, Inthata et al. [35] employed an ANN to model the chloride permeability of concretes containing ground pozzolans. The ANN model was verified using linear and nonlinear equations. The results indicate that the ANN demonstrates high prediction accuracy for evaluating the permeability compared to the linear and nonlinear methods. Similarly, the light gradient boosting algorithm and XGboost techniques were engaged by Alabdullah et al. [44] to estimate the rapid chloride penetration test. The model results showed that light GBM outperformed the XGBoost model in estimating the rapid chloride penetration test.

## 2. Significance of Research

Previous studies have revealed that incorporating SCMs in concrete might reduce the Dcl and avert the corrosion of steel reinforcement [45,46]. Chalee et al. [47] investigated the effects of the w/b ratio and fly-ash specific surface on the Dcl of concrete. The Dcl increased with a high w/b ratio [48]. However, few studies have investigated the influence of SCMs such as GGBFS and mix design concerning the chloride diffusion coefficient ML approach [35,36,49]. Therefore, this study could provide a comprehensive literature reference value for subsequent related requirements and has an excellent guiding significance for engineering practice, as the evaluation of durability-related properties requires extensive laboratory experiment, resources, and time consumption. This study aimed to comprehensively explore the capability of ML algorithms including ANN, ELM, SVM, and ANFIS in estimating the chloride diffusion of concrete containing SCMs. The best-developed model among the ML algorithms employed in this study was determined based on the reference index (RI) of the performance evaluation matrix.

## 3. Database Development

### 3.1. Input and Output Parameters

A high-quality dataset of 105 datasets were collected from the previous studies [46,50,51]. Each dataset consisted of eight input and one output variables, which included C_3_A content, W/B ratio, cement, fly ash, GGBFS, aggregate and water content, and specific surface. The Dcl was taken as the output parameter. Table 1 presents the initial descriptive statistics of the datasets. The descriptive statistics presented in Table 1 are the basic and most important information used to describe the dataset for the modeling task. The maximum, minimum, mean, and standard deviation of each input parameter and target parameter are presented. As shown in Table 1, the C_3_A content was 2 to 61.4% with a mean and standard deviation of 20.19% and 20.39%, respectively. Similarly, the descriptive statistic of all of the other parameters is described in Table 1. The mean value of chloride diffusion was 9.44 × 10^−12^ m^2^/s. Moreover, the skewness and kurtosis values revealed that the dataset is reliable for computational AI modeling since low skewness values were obtained in most input parameters.

### 3.2. Pearson Correlation Matrix

**Figure 1:**
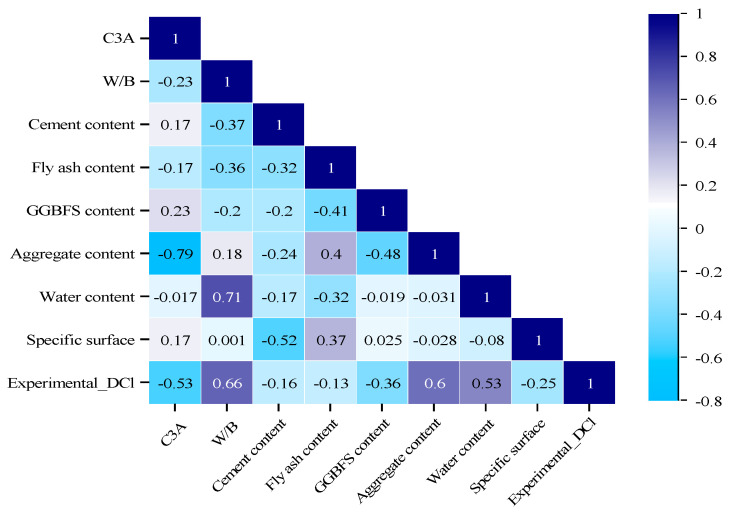
Correlation matrix of the variables.

Numerous studies have widely used many techniques for linear and nonlinear datasets to deal with irrelevant potential input variables in the modeling task because including the irrelevant variables in the artificial intelligence model reduces the estimation accuracy and increases the computational request [52]. Therefore, our study used the Pearson correlation matrix to explore the most relevant variables from the datasets, as depicted in Figure 1. The parameter relevance reduces as it is close to zero and increases to unity (−1 or +1). The unity values showed the perfect parameter. The positive value indicates a direct correlation between the input and target variable and vice-versa. As shown in Figure 1, the water/binder ratio and aggregate content appeared to be the most dominant variables, with a correlation value of 0.66 and +0.60 for predicting the Dcl of concrete containing SCMs, respectively. Conversely, C3A was negatively correlated with output parameters with high values.

### 3.3. Mutual Information

The MI technique evaluates the statistical dependency between two independent variables. The MI value = 0 indicates no statistical dependence between the two variables. The reliance increases with increasing distance from zero [53].

(1) MI(x,y)=f(x)+f(y)−f(x,y)

where *f*(*x*) is the entropy function of *x*, and the joint entropy between the two parameters *x* and *y* is represented by *f* (*x*, *y*) and can be expressed in Equation (2).

(2) $$f(x,y)=-\sum x\in X\sum y\in Y.P_{XY}(x,y)\log P_{XY}(x,y)$$

$P_{XY}(x,y)$ is the combined distribution of the parameters *x* and *y.*

**Figure 2:**
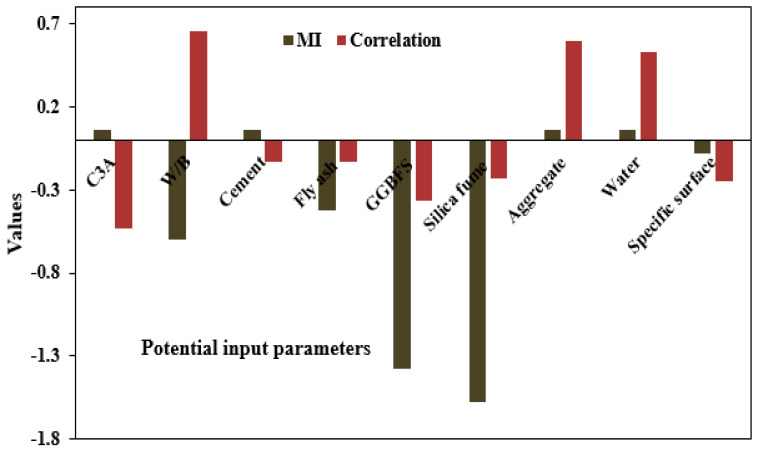
Determination of the relevant input variables using the MI technique.

Figure 2 shows the dependency of individual input variables using the MI approach. High-relevance input variables are achieved when the MI value is greater than 0. As seen in Figure 2, the C3A, cement, aggregate, and water content appeared to be the most relevant factors in terms of MI, with values of 0.0604 each. On the other hand, a negative relationship was obtained between the target parameter and W/B, fly ash, GGBFS, and fumed silica. 

Considering the two feature selection methods adopted in this study, the most relevant parameter revealed using Pearson correlation in the sensitivity analysis appeared to be the most sensitive parameter in modeling the coefficient of diffusion of concrete containing SCMs. Using linear and nonlinear measures to choose the best input parameters for the modeling work is crucial based on the findings from the feature selection technique. The AI models should incorporate linear and nonlinear interaction parameters with the target variables to capture both the linearity and nonlinearity patterns of the process or data.

## 4. Machine Learning Technique

ML is an extensively used method for solving numerous engineering problems including classification and regression problems; ML establishes prediction according to the input data and learning type with the help of defined architecture. This study used four ML algorithms to train 105 databases obtained from the literature to estimate the Dcl of concrete containing SCMs. All of the models were trained on MATLAB version R2019b.

### 4.1. Normalization

**Figure 3:**
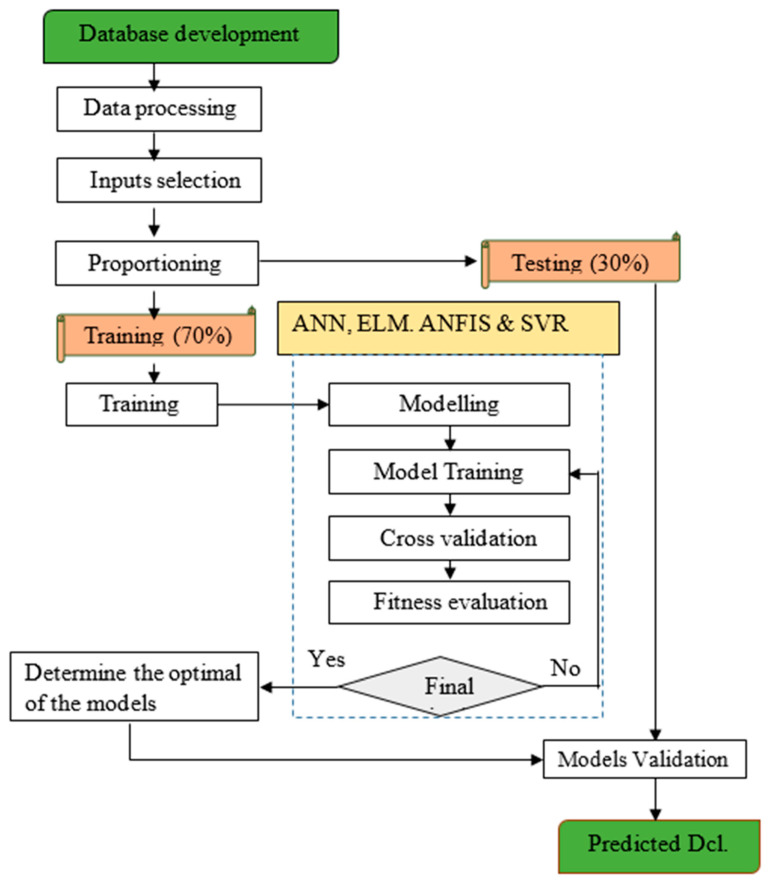
The flowchart of the developed models.

Equation (3) was used to normalize the dataset of concrete containing SCMs. The dataset was converted to the whole dataset with a common scale to reduce redundancy and enhance integrity before modeling. The normalization practice minimizes the inconsistency in the dataset and enhances model performance [25,54]. The methodology flowchart of the modeling is depicted in Figure 3.

(3) $$y_{norm}=\frac{y-y_{\min}}{y_{\max}-y_{\min}}$$

where *y_norm_* is the normalized data value *y*; *y*_min_ is the minimum measured data; and *y*_max_ is the maximum measured data.

### 4.2. Artificial Neural Network (ANN)

The ANN effectively simulates a complex task with hidden layers in the training phase. The ANN used data to create a relation between the dependent and target parameter [55]. The ML algorithm resembles a biological neural network in structure and functionality. The neural network model is more robust and relevant in practically all areas of engineering, science, and neuroscience [56]. Backpropagation (BP) neural networks are the most widely used neural network due to their simplicity [57]. The neural network comprises artificial neurons connected and includes several levels such as an input layer, at least one hidden layer, and an output layer. The neural network’s fundamental processing units are called nodes. To produce the output for the neurons, the inputs are multiplied by a modified weight and then sent to the transfer function [58]. The activation function converts the sum of weighted input and bias. The logistic and hyperbolic tangents are the commonly utilized activation functions given in Equations (4) and (5).

(4) $$f(x)=\left(\frac{1}{1+e^{-r}}\right)$$

(5) $$f(h)=\left(\frac{e^{h}-e^{-h}}{e^{h}+e^{-h}}\right)$$

**Figure 4:**
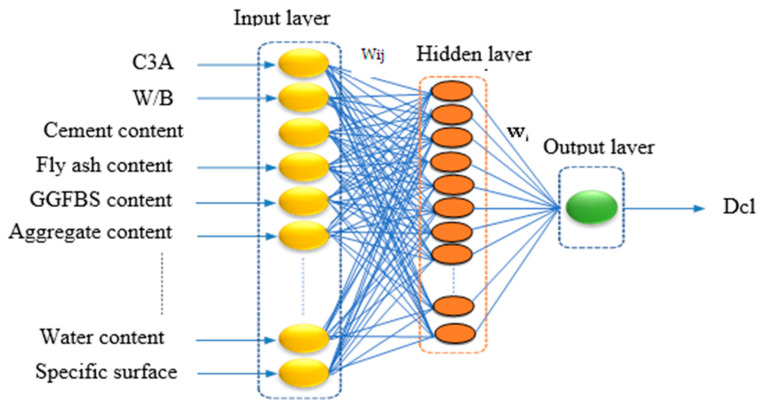
Structure of the ANN model.

Subsequently, the output *K_j_* of the neuron (*j*) in the *i*th layer is expressed in Equations (6) and (7).

(6) $$Y_{j}=\sum_{i=1}^{n}\left(c_{ij}x_{i}+b_{i}\right)$$

(7) $$K_{j}=f(Y_{j})$$

where *Y_j_* is the neuron; *j* is the activation value for the *i*th layer; *r*_i_ is the *i*th input vector of n input; *f*(*x*) is the activation function; *c_ij_* denotes the weight of the *i*th input; and neuron *j* and *b_i_* are the bias term. The feed-forward network, as the name suggests, propagates information forward. Backpropagation (BP) algorithms are widely used to train a neural network [59]. However, to address the shortcomings of backpropagation algorithms, Levenberg–Marquardt has been developed as a second-order variant. It primarily employs the gradient steepest descent approach for training. The Levenberg–Marquardt algorithm improves the weights during the training phase by relating the stability of the steepest descent method with the speed advantage of the Gauss–Newton algorithm [60]. After several trials, the number of neurons with the highest determination coefficient and the MSE between the predicted and observed data were selected as the optimum number of hidden neurons [56]. The ANN model structure is shown in Figure 4.

### 4.3. Extreme Learning Machine (ELM)

**Figure 5:**
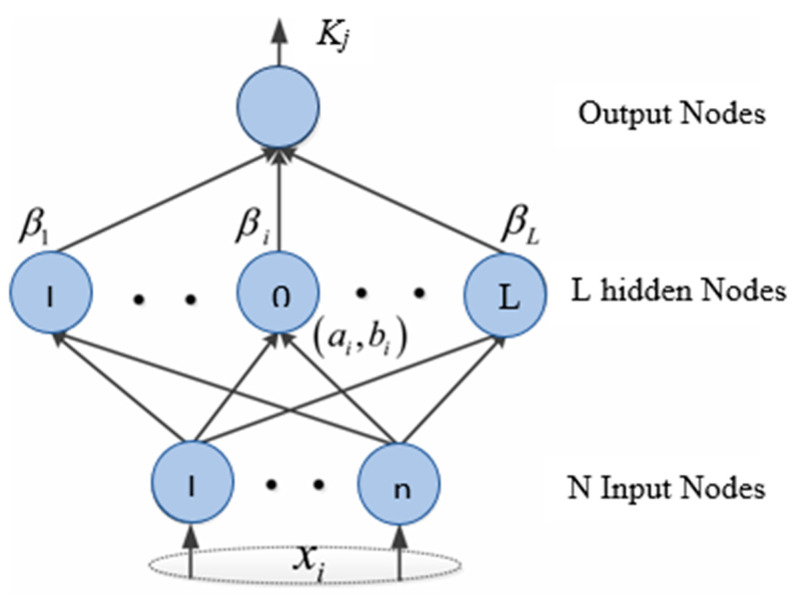
The architecture of the ELM model.

**Figure 6:**
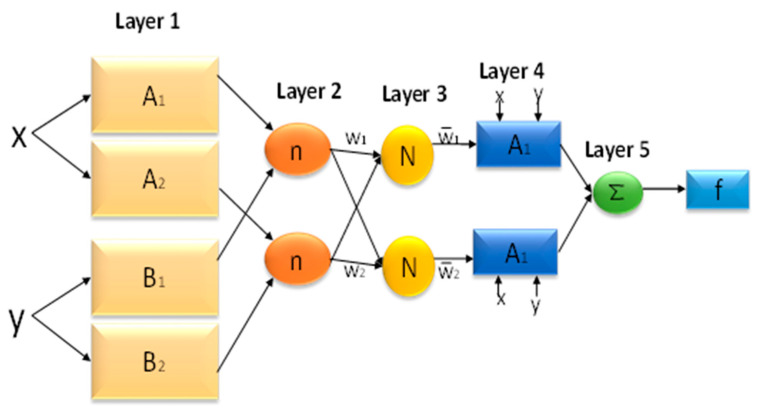
Architecture of the ANFIS model.

An extreme learning machine is a robust learning tool for a single-hidden layer feed-forward neural network (SLFN), as presented in the structure diagram depicted in Figure 5. Analytical calculations are used to evaluate the output weights in ELM, and hidden node weights and biases are created at random, leading to the enhancement in the learning speed of the network [61,62]. In the ELM, several hidden node parameters such as input weights, bias, and impact factors are created randomly. In Figure 6, *x*_j_ represents the input parameter, and L is the number of parameter vectors in the extreme learning machine feature space acquired by parameter mapping [63] and linear variable solving [64]. ELM has three properties [65] compared with conventional ANN:

(i) The linking weights and thresholds are artificially set, which can be adjusted after setting. However, backpropagation neural networks require frequent adjustment of the two values. Therefore, ELM shortened the execution time by 50 percent in comparison with BPNN.
(ii) The only number of the neuron parameter in SLFN requires adjustment.
(iii) The ELM obtains the solution by solving equations to evaluate the target weight *β*, without an iteration of fine-tuning.

In the ELM principle, there exist samples $\left(x_{i},z_{j}\right)$ where

$$x_{i}={\left[x_{i1},x_{i2},x_{i3}\ldots \ldots ,x_{in}\right]}^{T}\in P^{n},z_{i}={\left[z_{i1},z_{i2},z_{i3}\ldots \ldots ,z_{in}\right]}^{T}\in P^{m}$$

The single implicit layer model with L implicit layer nodes is given in Equation (8)

(8) $$K_{j}=\sum_{i=1}^{L}\beta f(Q_{i}.X_{j}+b_{i})$$

where $Q_{i}={\left[Q_{i1},Q_{i2}\ldots Q_{in}\right]}^{T}$ describes the input weight; $\beta_{i}$ is the weight of the output; and $b_{i}$ represent the bias of the *i*th hidden layer node; $x_{j}$ is the set of features of one sample, as expressed in Equation (9).

(9) $$\sum_{j=1}^{N}\left|K_{j}-z_{i}\right|=0$$

There exists $\beta_{i}$,$Q_{i}$, and $b_{i}$ so Equation (10) can be formed:

(10) $$\sum_{i=1}^{L}\beta_{i}f(Q_{i.}+b_{i})=z_{i},j=1,2,N$$

### 4.4. Adaptive Neuro-Fuzzy Inference System (ANFIS)

A robust hybrid technique can solve the complicated relationship, which combines ANN and fuzzy systems [66]. The ANFIS uses the neural network’s capacity for learning with the benefits of a fuzzy rule-based system that may consider prior observations when classifying data. Unlike a trained neural network, a fuzzy system is constructed using fuzzy logic definitions. Figure 6 shows the structure of the ANFIS, containing five layers built like a multi-layer feed-forward neural network for mapping inputs with output parameters. Moreover, multilayer feed-forward (MLFF) neural networks, which map inputs to output parameters, are created by combining fuzzy logic systems and neural networks with the ANFIS [67]. ANFIS is effective in coping with the uncertainty of human behavior due to its adaptability, flexibility, and capacity to manage enormous amounts of noisy data from complex and dynamic systems [68]. Similar to other soft computing techniques, ANFIS has its shortcomings; however, the tool is most suitable for inference systems like the Takagi–Sugeno and Mamdani. ANFIS rule systems are classified into Mamdani, expressed as a mathematical function, and Takagi–Sugeno, expressed as a linguistic variable. While the Mamdani FIS rule requires defuzzification, the Sugeno does not require any defuzzification process [69].

### 4.5. Support Vector Regression (SVM)

SVM is a robust, innovative supervised machine learning algorithm generally used in solving classification and regression problems. The SVM is called support vector regression (SVR) for regression problems. For the set of the dependent variable (*x*) and the independent variable (*y*), a relationship can be developed based on the following equation

(11) f(x)=ω·φ(x)+b

where *ω* is the weight vector; *φ*(*x*) is the nonlinear function that maps the inputs space vector *x* into high dimensional feature space; and *b* is a bias.

Equation (11) shows that the SVM employs linear regression in a high dimensional feature from the input space by nonlinear mapping. Kernel functions, namely, polynomial (Poly), linear (Lin), and radial basis function (RBF), are commonly used to convert input data into a high-dimensional feature space. This study used RBF kernel due to its high performance. By minimizing the error function, the parameters *ω* and b can be estimated as

(12) $$\frac{1}{2}{\left\|\omega \right\|}^{2}+C\frac{1}{n}\sum_{1}^{n}L_{e}{(f(x}_{i}{),y}_{i})$$

(13) $$L_{\varepsilon }(f(x),y)=\{\begin{array}{ll} 0 & \;\mathrm{if}\;|f(x_{i})-y_{i}|\;<\;\varepsilon \\ \mathrm{if}\;|f(x_{i})-y_{i}|-\varepsilon \; & \;\mathrm{otherwise} \end{array}$$

where 1/2 ‖*ω*‖^2^ is the regularization term; *C* is the penalty parameter; *L_e_* is the e-sensitive error function; *f*(*x_i_*) is the predictive value; *y_i_* is the target value; and *ε* is the tube size of SVM. Equation (14) can be used to train the SVM using the RBF kernel.

(14) $$K(x_{i},x_{j})=\exp(-\gamma {\left\|x_{i}-x_{j}\right\|}^{2}),\;\gamma >\;0$$

The parameters *ε*, *γ*, and *C* were appropriately set to obtain an accurate and satisfactory performance. These parameters were obtained from trial and error. 

### 4.6. Hyperparameter Tuning and Cross-Validation

**Figure 7:**
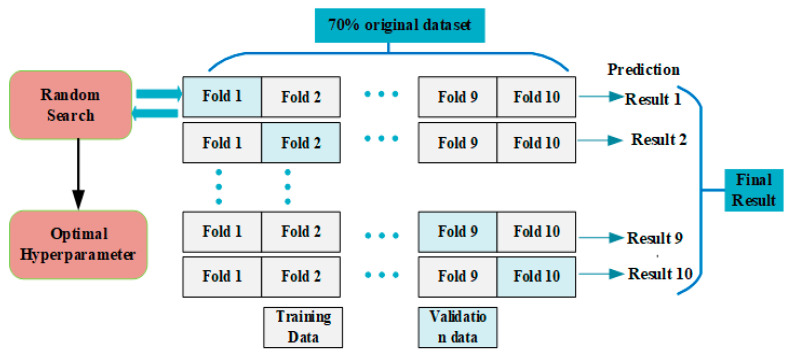
Hyperparameter tuning.

Hyperparameter tuning is an optimization system on top of model training to examine for hyperparameter outcomes in a lower error. Although the hyperparameters can be obtained using a manual Bayesian optimization, grid, and random search were employed to automate the tuning process. This study used a random search to tune and select the hyperparameters. Cross-validation is an effective technique used to prevent the problem of overfitting and is set when the turning hyperparameter is on a small database. Therefore, tenfold validation, as depicted in Figure 7, was adopted in our study. The dataset was proportioned into 70 for training and 30 for testing. The training dataset was divided into ten subsets, and one subset was considered for testing the trained model for individual iteration. The *i*th part of every iteration (*i* = 1, 2, 3, 4, 5…, 10) was considered the test data, and the remaining dataset was taken as the training phase. The turning step was utilized to evaluate the best ANN algorithm in Table 2.

### 4.7. Model Evaluation Metrics

This study employed the most widely used performance indicator to check the prediction results of the three predictive models. These metrics include the coefficient of determination (R^2^), mean absolute error (MAE), root mean square error (RMSE), mean absolute percentage error (MAPE), and reference index (RI). The equation and description of each indicator are presented in Table 3, where *y_i_* and *p_i_* are the observed and predicted values; ȳ is the mean of the observed data; and *n* is the number of observations.

## 5. Result and Discussion

### AI-Based Model Results

This study used MATLAB (2019b) toolbox to develop the ML model. The model’s validation was performed using a 10-fold cross-validation system [22,52,72]. The models used were the training and testing datasets. Table 4 presents the performance of the model algorithm using the evaluation metrics. The ML model accuracy was evaluated and compared using the performance evaluation criteria, which include R^2^, MAE, RMSE, MAPE, and RI. The lower MSE, RMSE, MAE, and MAPE values showed a high prediction accuracy, and high R^2^-values indicate good performance. As shown in Table 4, all of the ML models estimated the Dcl with high accuracy in both the training and testing stages with R^2^ values greater than 90%. Moreover, SVM appeared to be the best model for estimating the Dcl of concrete modified with SCM with the coefficient of determination value (R^2^) of 0.959 and 0.958 at the training and testing phase, respectively. The normalized RI values were used to validate and rank the accuracy of the three ML models since the other indicators may not reflect the overall error of the models. It can be seen that the SVM performed best with a RI value of 0.930 and 0.803 for the training and testing stage, respectively. The ANN came in second, with a RI value of 0.900 and 0.801 for the training and testing, respectively. Liu et al. [36] described the ANN model as an effective algorithm to ascertain the differences in the observed dataset and is specifically helpful in estimating the chloride resistance of reinforced concrete structures. The results of the developed models were compared with the available AI models, as shown in Table 5. The R-value shows that the developed model performed better than Nhat et al. [37] and similar to Ahmet et al. [34] and Parichatprecha et al. [73].

**Figure 8:**
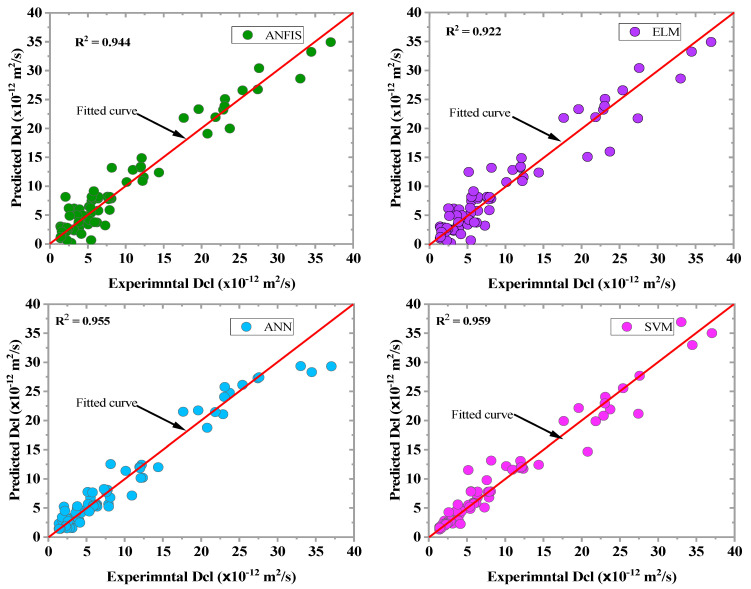
Observed vs. predicted plots of the ML models for the training phase.

**Figure 9:**
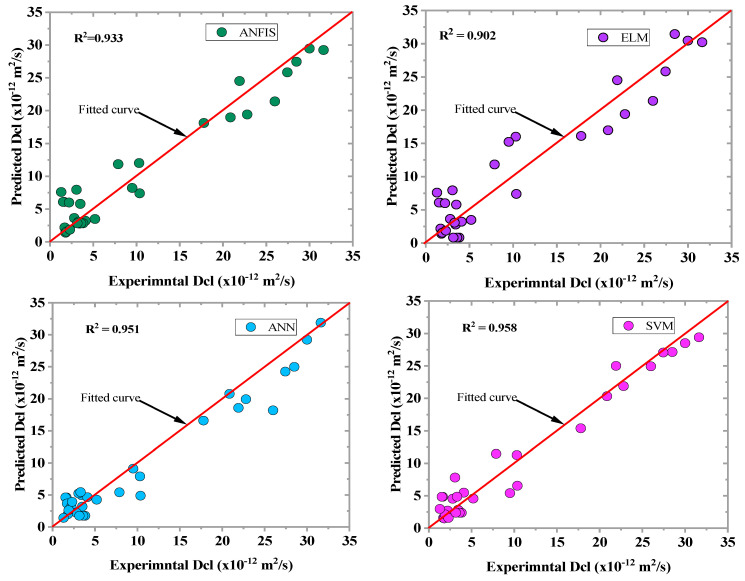
Predicted vs. measured plots of the ML models in the testing phase.

The scatter plots between the observed and predicted values in the training phase are depicted in Figure 8. The data were converged along the fitting line of the developed ML model’s plots, translating to higher goodness of fit. By comparing the R^2^ and RI values of the developed models at the two modeling stages, as shown in Figure 8 and Figure 9, the prediction accuracy of the ML algorithms was checked. All of the ML algorithms predicted chloride diffusion with high accuracy. The SVM model demonstrated the highest prediction accuracy with a R^2^ value of 0.959, which was greater than that of the ANN, ANFIS, and ELM models by 0.42%, 1.17%, and 5.8%, respectively.

**Figure 10:**
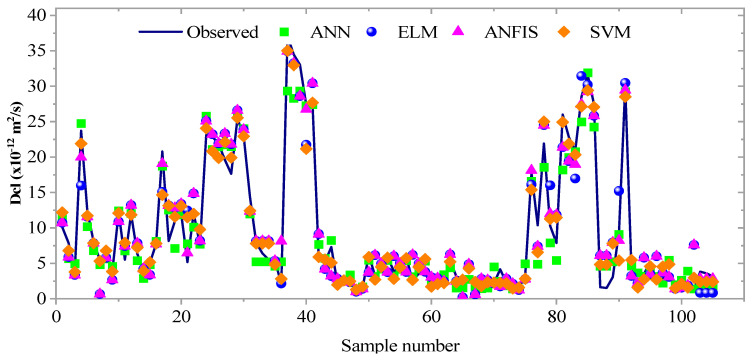
Predicted vs. measured plots of ML models in the testing phase.

Similarly, the SVM model revealed the highest prediction accuracy in the testing phase compared to other models (ANN, ANFIS, and ELM), as shown in Figure 9. Moreover, ANN appeared to be the second-best model for predicting the Dcl incorporated with SCMs. The result showed that the Dcl of concrete could be analyzed with minimum error using the ML algorithms (ANN, ELM, SVM, and ANFIS). The scatter plot of the measured vs. predicted values at the testing stage is shown in Figure 10. The degree of agreement between the measured and predicted values showed the efficacy and accuracy of each of the three ML models in determining the nonlinear relationship between the eight input parameters and the chloride diffusion coefficient. 

**Figure 11:**
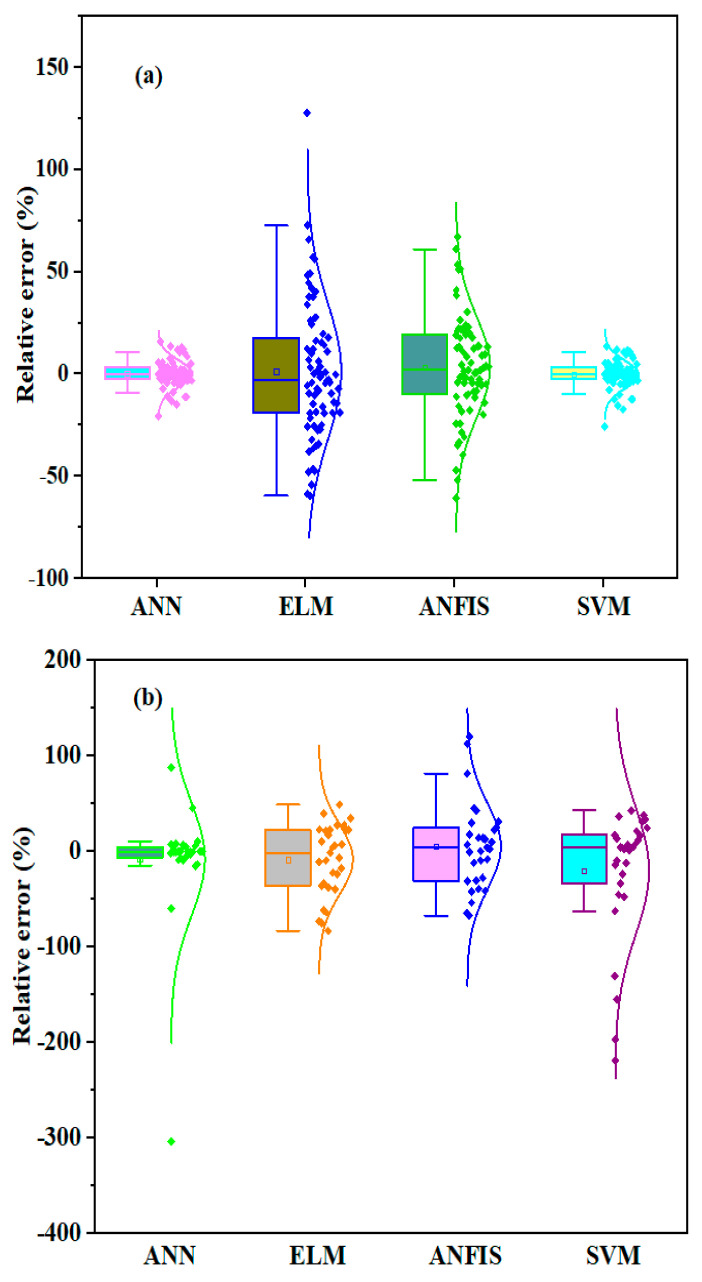
Relative error distribution for the developed model using Boxplot at (**a**) training and (**b**) testing phase.

The accuracy of the ML model was assessed using the relative error distribution obtained from each model. Therefore, Boxplot was used to compare the relative error distribution at the two modeling phases, as depicted in Figure 11. The SVM and ANN models (Figure 11a) demonstrated the best performance, exhibiting the lowest maximum and minimum relative error distribution in predicting the Dcl in the training phase. The SVM and ANN model’s first quartile (Q1) and third quartile (Q3) values were less than 5%. Moreover, the relative error distribution revealed by all of the ML developed was relatively lower in the training phase compared to the testing phase, as shown in Figure 11b. Figure 11b shows that the lowest relative error distribution was also obtained in the SVM models during the training phase. The minimum and maximum relative error distributions in Q1 and Q3 were −4.5% and 5.7%, respectively.

**Figure 12:**
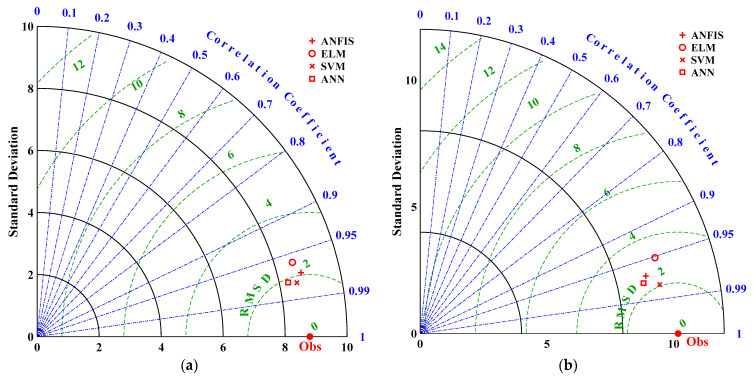
Taylor diagram comparing the overall model’s performance for (**a**) training and (**b**) testing phase.

To further evaluate the effectiveness of the developed ML model for predicting the diffusion coefficient, the Taylor diagram was drawn using three statistical metrics including R^2^, root mean square difference (RMSD), and STD. Figure 12 indicates that the ANN had the lowest RMSD and the highest R-value. However, all the ML models could accurately predict the chloride diffusion coefficient with RMSE, *R*, and STD closer to that of the overall actual data. The azimuthal point describes the correlation between the measured and predicted values. The RMSE is related to the point between the measured and predicted field; when the correlation increases, the RMSE value decreases. Thus, the standard deviation trend increases with an increase in the radial distance measured from the origin [74]. The model is considered an excellent model by reference point when its R^2^ value = 1 [75]. However, overestimation might occur if the predicted standard deviation is higher than that of the observed values and vice versa. Thus, a STD approach to the standard deviation of the actual data is often required. 

## 6. Conclusions

In this study, a database from past studies was collected to develop the ML model including artificial neural network (ANN), extreme learning machine (ELM), adaptive neuro-fuzzy inference system (ANFIS), and support vector machine (SVM) to predict the chloride diffusion coefficient (Dcl) of concrete containing SCMs. Sensitivity analysis using PC and the mutual information technique was employed to explore the most relevant input variables. Five evaluation matrices were used to assess the model’s performance including the MSE, RMSE, R^2^ MAE, and RI. To summarize the findings, the following conclusions are highlighted:

1. The sensitivity analysis using the Pearson correlation matrix showed that the water/binder ratio is the most relevant parameter for estimating the Dcl of concrete containing SCMs, considering the linear and nonlinear pattern of the dataset. On the other hand, the cement, aggregate, and water content appeared to be the most relevant parameters with a MI value greater than zero.
2. The four developed ML models estimated the chloride Dcl of concrete with high accuracy at the two modeling phases. Moreover, the highest prediction accuracy was obtained in the SVM models with R^2^ values of 0.959 and 0.958 in the training and testing stage, respectively.
3. The study’s findings prove the single AI-based model’s ability to estimate the chloride diffusion coefficient of the concrete incorporated with SCMs with higher performance. Although the established models revealed high prediction accuracy, it is recommended that recent and advanced machine learning algorithms such as hybrid and ensemble models are employed to evaluate the chloride diffusion coefficient containing supplementary cementitious materials.
4. Investigating the durability-related properties of steel-reinforced concrete caused by the chloride diffusion coefficient is essential. It can provide a comprehensive literature reference value for subsequent related requirements and guide engineering practice, as the evaluation of durability-related properties requires extensive laboratory experiments, resources, and time consumption.

## Figures and Tables

**Table 1:** Initial statistics of the dataset.

Direction	Description	Units	Max	Min	Mean	STD	Skewness	Kurtosis
Inputs	C_3_A Content	%	61.4	2	20.19	20.39	0.91	−0.45
	W/B	-	0.83	0.26	0.42	0.11	1.28	2.10
	Cement content	kg/m^3^	660	120	302.35	83	1.54	3.21
	Fly ash content	kg/m^3^	275	0	84.40	81.60	0.77	−0.19
	GGBFS	kg/m^3^	275	0	27.72	62.74	2.11	3.58
	Aggregate content	kg/m^3^	1997	853	1454	411	−0.2	−1.86
	Water content	kg/m^3^	193	133	167.01	11.39	−0.04	0.99
	Specific surface	cm^2^	6510	0	2581.83	1639.85	−0.34	−1.16
Output	Diffusion coefficient Dcl	×10^−12^ m^2^/s	37.04	1.29	9.44	9.24	1.31	0.59

**Table 2:** Model hyperparameter.

Model	Hyperparameters	Values
ANN	Hidden layer sizes	10
	Activation function	tanh
	max_iter	1000
	Tolerance	0.0001
SVM	Regularization parameter C	1.2
	Kernel function	Gaussian
	Gamma	1.32
ELM	Hidden layer sizes	10
ANFIS	Cluster no.	12
	Membership function	Takagi–Sugeno

**Table 3:** Evaluation metrics.

Metrics	Equations	Descriptions
R2	$1-\frac{\sum_{i=1}^{n}{\left(y_{i}-p_{i}\right)}^{2}}{\sum_{i=1}^{n}{\left(y_{i}-\bar{y}\right)}^{2}}$	The R2 is the model’s fitness in estimating the observed data. It is within the range of 0 to 1; better performance is obtained when the R2-value approaches 1 [70].
MAE	$\frac{1}{n}\sum_{i=1}^{n}|y_{i}^{\prime }-y_{i}|$	The MAE metric measures the absolute difference between the observed and predicted values, but it cannot reflect the degree of error in relation to the actual value.
RMSE	$\sqrt{\frac{1}{n}\sum_{i=1}^{n}{\left(y_{i}-p_{i}\right)}^{2}}$	RMSE describes the variation between the observed and predicted value. It always takes a positive value, and the minimum values indicate a better prediction.
MAPE (%)	$\frac{1}{n}\sum_{i=1}^{n}|\frac{p_{i}-y_{i}}{y_{i}}|\times 100$	MAPE demonstrates how well the model could estimate the observed values by expressing the percentage errors. The smaller percentage revealed a better prediction of the algorithm [71]
RI	$\frac{MAE+RMSE+MAPE}{3}$	Reference index (RI) is a function of three errors normalized to obtain the optimum performance

**Table 4:** Performance evaluation.

Models	Phase	R^2^	MAE	RMSE	MAPE	RI	Rank
ANN	Training	0.955	1.280	1.894	20.86	0.900	2
	Testing	0.951	1.905	1.63	40.96	0.801	
ELM	Training	0.922	1.790	2.459	31.00	0.846	4
	Testing	0.902	2.623	3.175	70.24	0.649	
ANFIS	Training	0.944	1.615	1.416	21.40	0.898	3
	Testing	0.924	2.152	2.800	62.55	0.690	
SVM	Training	0.959	1.065	1.789	14.80	0.930	1
	Testing	0.958	1.640	1.337	41.05	0.803	

**Table 5:** Comparison of the results with the existing AI models.

Reference	Technique	Specimen Type	Input Variables	Datasets	R
Nhat et al. [37]	Multi-gene genetic programming and multivariate adaptive regression splines	Mortar	4	132	0.95
Ahmet et al. [34]	ANN and ANFIS	GGBS-based concrete	4	162	0.98
Parichatprecha et al. [73]	ANN	HPC	8	86	0.98
Authors	ANN, ELM, ANFIS, and SVM	GGBS-based concrete	8	105	0.98

## Data Availability

Data obtained as described.

## Abbreviations

**Terms**	**Abbreviation**
AI	Artificial intelligence
ANN	Artificial neural network
ANFIS	Adaptive neuro-fuzzy inference system
ELM	Extreme learning machine
SVM	Support vector machine
Dcl	Chloride diffusion coefficient
SCMs	Supplemental cementitious materials
C_3_A,	Tricalcium aluminate
GGBFS	Ground granulated blast furnace slag
W/B	Water binder ratio
RI	Normalized reference index
R^2^	Coefficient of determination
MAE	Mean absolute error
RMSE	Root mean square error
MAPE	Mean absolute percentage error
ML	Machine learning
MI	Mutual information
BP	Backpropagation
MLFF	Multilayer feed-forward
RBF	Radial basis function
